# The texture of collagen and immunoexpression of PRAME in dysplastic nevus syndrome lesions: relationship with melanoma^⋆^

**DOI:** 10.1016/j.abd.2022.02.002

**Published:** 2022-11-08

**Authors:** Paula Regina Martins Costa, Gislaine Vieira-Damiani, Rafael Fantelli Stelini, Leonardo Ávila Ferreira, Maria Letícia Cintra, Fernanda Teixeira

**Affiliations:** a Department of Pathology, Faculdade de Ciências Médicas, Universidade Estadual de Campinas, Campinas, SP, Brazil; b Instituto Federal de Educação, Ciência e Tecnologia de São Paulo, Capivari, SP, Brazil; c Department of Dermatology, Faculdade de Ciências Médicas, Universidade Estadual de Campinas, Campinas, SP, Brazil

**Keywords:** DN, Dysplastic Nevi, DNHG, Dysplastic Nevi With High Grade Atypia, CM, Cutaneous Melanoma, DNLG, Dysplastic Nevi With Low/Moderate Grade Atypia, DNS, Dysplastic Nevus Syndrome

Dear Editor,

Although Dysplastic Nevi (DN) present a degree of cytoarchitectural disorder, it is generally not difficult to distinguish them from cutaneous melanoma (CM). However, in some, this distinction can be difficult.^1^ Patients with numerous DN^2^ and those with DN with high-grade atypia (DNHG)^3^ are more likely to develop CM. These patients need close follow-up and any changing lesion must be excised to rule out malignancy, previously misdiagnosed as a dysplastic nevus. One of the remarkable microscopic findings of DN is fibroplasia of the papillary dermis.^4^ PRAME (PReferentially expressed Antigen in MElanoma) is an antigen associated with the majority of primary and metastatic cutaneous and uveal melanomas, with the exception of desmoplastic melanomas.^5^ The authors studied the density and texture of the collagen underlying DN, excised from 15 patients with dysplastic nevus syndrome (DNS), in the 1994‒2019 period, and the expression of PRAME in their cells.

## Methods

**Figure 1:**
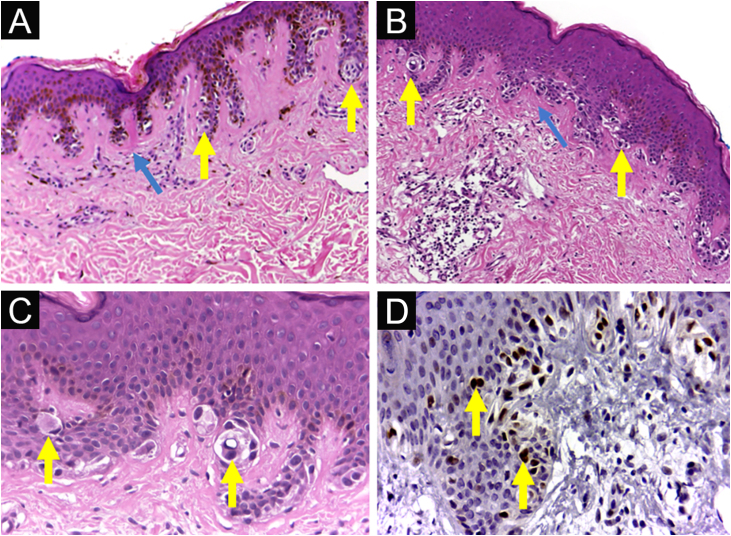
**Dysplastic nevus syndrome:** (A) Classical Dysplastic Nevus (CDN): regular epidermal hyperplasia, small clusters of melanocytes at the dermoepidermal junction (yellow arrows) and papillary dermis fibroplasia (blue arrow); (B, C) DN with High-Grade histological atypia (DNHG): irregular epidermal hyperplasia, melanocyte aggregates of varying volumes, in varied distribution and with moderate to marked multifocal cytological atypia (yellow arrows) and papillary dermis fibroplasia (blue arrow); (D) DN with High-Grade histological atypia (DNHG): nuclear immunoexpression of the PRAME antigen in the melanocytes present at the dermoepidermal junction. (A‒C) Hematoxylin & eosin; (D) immunohistochemistry; ×100 (A, B) and ×400 (C, D).

**Figure 2:**
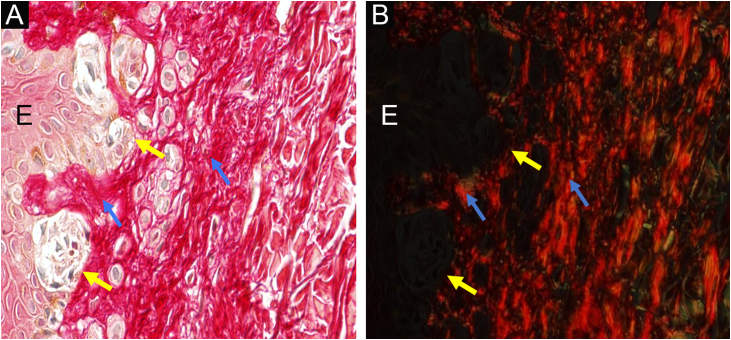
Dysplastic nevus syndrome: classical dysplastic nevus- on the left, the epidermis (E) shows melanocyte aggregates at the dermoepidermal junction (yellow arrows) and, on the right, the papillary dermis under the nevus is indicated by blue arrows ×400. Picrosirius red, without polarization (A) and under polarization (B).

Institutional Review Board approval (no. 3,548,935) was obtained. The patients were regularly monitored at the Dermatology Outpatient Clinic. From the nevi that were excised from these patients during this period, 56 were diagnosed histologically as junctional DN, and had enough remaining embedded tissue for additional sections. Nevi were diagnosed with low/moderate (n = 32, DNLG) or moderate/severe (n = 24, DNHG) grade/cytoarchitectural disorder (Fig. 1A‒D). Each specimen was studied in two ways: 1) By staining with picrosirius red and observation under polarized light on digitized images (Fig. 2), to assess collagen density and texture under the nevus, using ImageJ software (http://rsb.info.nih.gov/ij) to measure the contrast of the grey level co-occurrence matrix, second angular momentum, entropy and anisotropy, and 2) By conventional immunohistochemical methods, for its PRAME (Mab EPR20330; Abcam, #219650) expression, according to the method by Googe et al.^5^ For statistical analysis, the software used was the SAS System for Windows.

## Results

Nine of the fifteen patients were female, eight had a previous history of CM at some point in their life, 51/56 DN were diagnosed up to 40 years of age, and no lesion recurred after excision. Collagen under the DN of patients with a personal history of CM had significantly higher optical density values (p = 0.0259) compared to those without this precedent, denoting a more compact texture. DNHG (n = 24/56) had significantly lower contrast (p = 0.0140) and entropy (p = 0.0353) values compared to DNLG, reflecting greater collagen organization. These results confirm the greater predisposition of these DNS patients to CM. As Babacan et al. found by histochemical methods,^6^ it seems that the modulation of the extracellular matrix evolves in parallel with the cytoarchitectural disorder. PRAME was not overexpressed in DN from patients with DNS. The nuclear PRAME staining of DN melanocytes was categorized as absent in 51 DN and focally present^5^ in 5 DNHG lesions (Fig. 2). Googe et al.^5^ found only focal immunoreactivity for PRAME in just over 10% of nevi, including dysplastic ones.

## Conclusion

DNHG and DN with underlying compact texture appear to be markers of patients at increased risk of developing melanoma.

## Acknowledgements/ Funding sources

The antibody and further items used for the development of this work was purchased with the help of FAEPEX-Uncamp (Fund to support teaching, research and extension), Grant #2015/20. Paula R. M. Costa received a scholarship from CNPq/ Pibic (the National Council for Scientific and Technological Development). We reviewed the content of the manuscript, followed by Ms Diane Ellis, B.A. in education. Biostatistician Cleide Aparecida Moreira Silva, Research Committee, Biostatistics Division, Medical Sciences School, Unicamp, provided statistical consultation.

## Author’s contribution

Paula Regina Martins Costa: Study concept; data collection; writing of the manuscript.

Gislaine Vieira-Damiani: Analysis and interpretation, critical review.

Rafael Fantelli Stelini: Data collection; research guidance.

Leonardo Ávila Ferreira: Data collection; research guidance.

Maria Letícia Cintra: Data collection; writing of the manuscript; effective participation in the research guidance.

Fernanda Teixeira: Data collection; manuscript critical review; writing of the manuscript.

## Conflicts of interest

None declared.
